# Photocatalytic Hydrolysis—A Sustainable Option for the Chemical Upcycling of Polylactic Acid

**DOI:** 10.1021/acsenvironau.3c00040

**Published:** 2023-10-02

**Authors:** Antonia Garratt, Klaudia Nguyen, Alexander Brooke, Martin J. Taylor, Maria Grazia Francesconi

**Affiliations:** † School of Natural Sciences, Chemistry, University of Hull, Cottingham Road, Hull HU6 7RX, United Kingdom; ‡ Energy and Environment Institute, University of Hull, Cottingham Road, Hull HU6 7RX, United Kingdom; § School of Engineering, Chemical Engineering, University of Hull, Cottingham Road, Hull HU6 7RX, United Kingdom

**Keywords:** polylactic acid, plastic upcycling, photocatalysis, sustainability, low energy processes, depolymerization

## Abstract

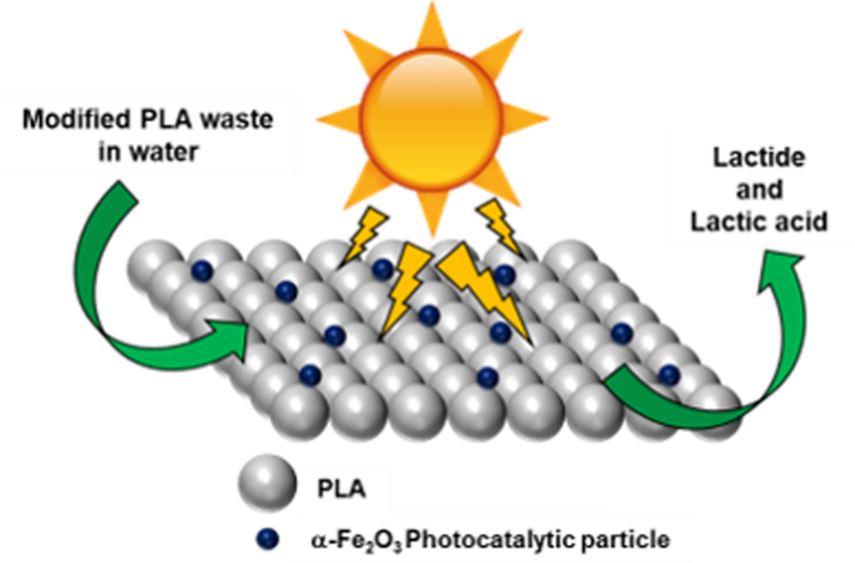

Plastic waste is a critical global issue, yet current strategies to avoid committing plastic waste to landfills include incineration, gasification, or pyrolysis high carbon emitting and energy consuming approaches. However, plastic waste can become a resource instead of a problem if high value products, such as fine chemicals and liquid fuel molecules, can be liberated from controlled its decomposition. This letter presents proof of concept on a low-cost, low energy approach to controlled decomposition of plastic, photocatalytic hydrolysis. This approach integrates photolysis and hydrolysis, both slow natural decomposition processes, with a photocatalytic process. The photocatalyst, α-Fe_2_O_3_, is embedded into a polylactic acid (PLA) plastic matrix. The photocatalyst/plastic composite is then immersed in water and subjected to low-energy (25 W) UV light for 90 h. The monomer lactide is produced as the major product. α-Fe_2_O_3_ (6.9 wt %) was found to accelerate the PLA degradation pathway, achieving 32% solid transformation into liquid phase products, in comparison to PLA on its own, which was found to not decompose, using the same conditions. This highlights a low energy route toward plastic waste upgrade and valorization that is less carbon intensive than pyrolysis and faster than natural degradation. By directly comparing a 25 W (0.025 kWh) UV bulb with a 13 kWh furnace, the photocatalytic reaction would directly consume 520× less energy than a conventional thermochemical pathway. Furthermore, this technology can be extended and applied to other plastics, and other photocatalysts can be used.

## Introduction

If plastic waste continues to grow following the current trend, the world will be facing an even more serious environmental crisis than it is now. Plastic waste volumes are predicted to increase to 460 million tons in 2030, raising an alarming worldwide concern.^1−3^ Notably, following the COVID-19 pandemic, the plastic crisis deepened with the increased use of single use plastic (SUP) in the form of plastic gloves, sheetings, masks, and many others to protect against the virus.^4^ It was estimated that, at the peak of the pandemic, over 1.6 million tons/day of plastic waste was being generated worldwide. This amounted to over 3.4 billion single-use face masks or similar items being discarded across the world daily.^4,5^ To tackle this waste, incineration, an energy intensive process was employed to transform plastic waste via combustion. However, it contributes largely to air pollution, as toxic gases and carbon dioxide are emitted.^6−8^

In an age of rising energy costs and a lack of energy security, be that through a staggered transition to renewable energy or supply issues due to conflict, it is our duty to find long-term low-energy consuming solutions to counteract plastic pollution, toward a much-needed green recovery, as well as the various Net Zero 2050 targets established in the wake of 26th United Nations Climate Change Conference of Parties (COP26) and reinforced at COP27. To reach these targets, sustainable waste management strategies must be used, specifically low/zero carbon emitters that can accommodate renewable energy inputs, unlike current operations which consume large amounts of energy and often have unregulated emissions. As a result, waste should be diverted from landfill sites which are major producers of greenhouse gases such as carbon dioxide and methane. Much of the ancestral plastic waste takes decades to decompose, with examples being polypropylene and polyethylene that require 10–20 years,^9,10^ whereas polyvinyl chloride waste residues can take far longer, >400 years.^10^

Polylactic acid (PLA) is a plastic material derived from lignocellulosic waste matter such as sugar cane, rice, wheat, potatoes and corn, and is becoming an attractive sustainable polymer choice, in comparison to petroleum-derived polymers.^11^ PLA requires up to 55% less energy to be produced than petroleum-derived polymers, and at the end of life, it decomposes often through composting, liberating CO_2_, thus avoiding prolonged residence in landfills, albeit leaching lactic acid into soil and groundwater.^11^ PLA is currently used in the medical industry, for tissue engineering or regenerative medicine, cardiovascular implants, drug carriers, orthopedic interventions, cancer therapies, skin/tendon healing, medical tools, and other equipment. PLA is also used in 3D printing, which played a crucial role during the COVID-19 global pandemic for providing personal protective equipment (PPE) and ventilator modifications.^12^ Due to PLA’s biodegradability, processes such as composting and anaerobic digestion are thought to be sustainable approaches toward the upcycling/handling of PLA at the end of its operational life. However, PLA has proved far less degradable than other biodegradable polymers, and this slow decomposition hinders PLA’s wider applicability.^13^ The degradation of PLA in the environment is catalyzed by microorganisms, and studies on their abundance over a range of different environments found that PLA-degraders are not widely distributed and thus the complete degradation of PLA in soil is slow, as well as the initiation itself.^14^

Hence, it is of paramount importance to find sustainable approaches to accelerated decomposition of PLA or, better, approaches to recycling and upcycling of PLA. Chemical recycling is earmarked as a sustainable avenue to make PLA more commercially attractive, as it is a form of recycling that creates value by turning waste back into chemical components. In the chemical recycling of PLA, the end goal is depolymerization, i.e., to convert the polymer to its monomer lactic acid or, better, lactide. Lactide, a cyclic lactic acid dimer, is the vital intermediate in the preparation of PLA via ring opening polymerization (ROP, the method preferred by industry). Lactic acid is polymerized to form oligomers (prepolymers), and then it is used to prepare lactide, via thermal unzipping depolymerization.^14^

The whole process for the formation of lactide requires complex and highly energy consuming steps.^15^ To design a sustainable depolymerization approach, we have exploited the slow natural process of the degradation of PLA to lactic acid and CO_2_, namely via hydrolysis and photolysis.^16^ Hydrolysis is the first step of a multistep process, leading to full decomposition of the polymer. When PLA is exposed to UV light, for example in outdoor applications, it is subject to decoloration and fracturing, leading to its decomposition. Our green approach to chemical upcycling of PLA combines hydrolysis and photolysis, where the plastic is irradiated in water under controlled conditions so that degradation pathways can be isolated and selective products can be acquired. This was carried out by embedding a photocatalyst (α-Fe_2_O_3,_) into the PLA matrix and then exposing the composite material to UV light in the presence of water. We named this process photocatalytic hydrolysis. As the photocatalyst we used α-Fe_2_O_3_, a semiconductor and widely investigated photocatalytic material for water splitting due to its low band gap (∼2.2–2.6 eV), minimal cost, relatively low toxicity, and abundancy, despite limitations such as short carrier lifetime (∼10–12 μs).^17^ Although Fe_2_O_3_ is used for medical applications as well as the proposed catalysts, there is a level of toxicity associated with nanoparticles <5 nm, with a lethal dosage of 100 mg/kg. However, nanoparticles >9 nm have been found to exhibit no obvious toxicity.^18^

## Experimental Section

### Materials Synthsis

#### α-Fe_2_O_3_ Particle Preparation

Iron(II) oxalate (0.4976 g, 3.11 × 10^–3^ mol) (Sigma-Aldrich, 99%) was heated to 500 °C at 1.0 °C/min, holding for 4 h under static air using a Carbolite CWF 1200 furnace.

#### Preparation of α-Fe_2_O_3_/PLA Composite

PLA Ribbon (RS PRO – PLA 1.75 mm) (0.7 g) was dissolved in 25 mL of dichloromethane (VWR Chemicals, ≥99.8%) with the addition of sonication using a Ultrawave U100H PACKED sonicator for 1 h to fully dissolve PLA. α-Fe_2_O_3_ powder (0.07 g) was added gradually into the solution under continuous stirring to produce a 10:1 PLA/photocatalyst ratio by weight. The mixture was added dropwise onto a glass slide placed in an Ossila spin coater while spinning at intervals of 200 rpm for 1000 s. This process was repeated until a composite of ∼30 mg was produced.

All catalyst characterization, reaction analysis and relevant instrument information are found in the Supporting Information.

## Results and Discussion

**Figure 1:**
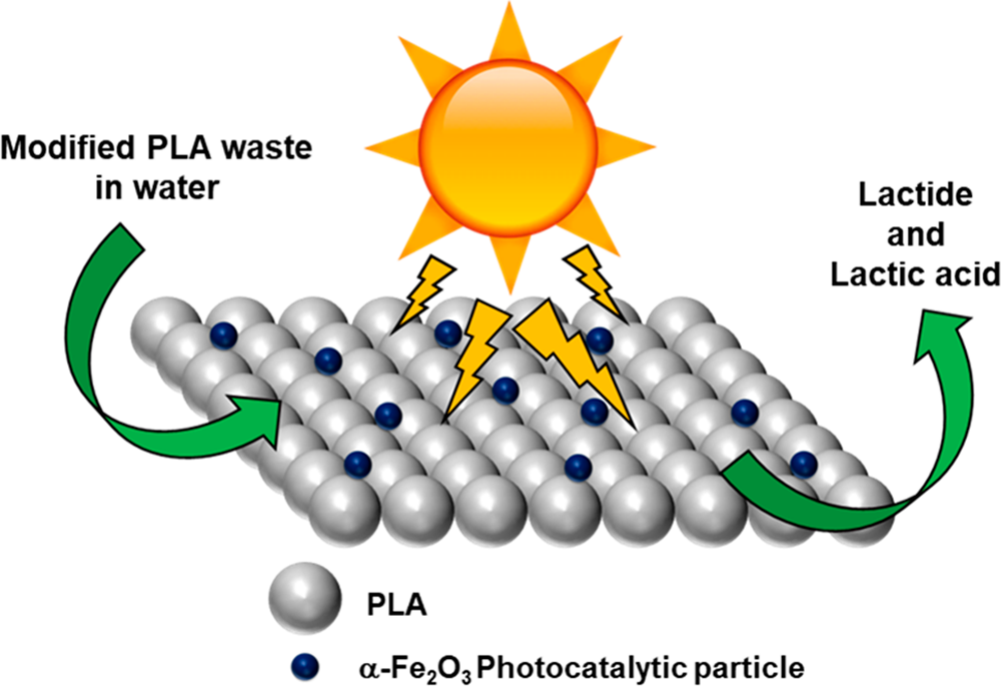
Schematic of the main steps involved in the photocatalytic hydrolysis of α-Fe_2_O_3_/PLA.

A schematic diagram of the steps involved in photocatalytic hydrolysis is shown in Figure 1. After waste PLA ribbon was dissolved in dichloromethane (DCM), α-Fe_2_O_3_ particles were dispersed into the PLA polymer matrix (∼10 wt %) to create a composite, α-Fe_2_O_3_/PLA (DCM). Samples of PLA without the photocatalyst (Raw PLA and Raw PLA (DCM)) were all subjected to the same photocatalytic hydrolysis parameters. The light source was a UV lamp with a wavelength of 254 nm and 25 W. The irradiation time was 90 h. It has been previously reported that accelerated photoaging of PLA films was performed in air at 60 °C with four 400 W mercury lamps for 100 h.^19^ In our work, only one UV lamp was used with a 25 W power for 90 h; this has been found to be sufficient to show the starting of the controlled decomposition process. We deemed this a suitable compromise to show proof of concept that a low-energy and experimentally uncomplicated process can depolymerize PLA into its most valuable monomer, lactide. Furthermore, for deployment as a future technology that can be used out of the laboratory, photocatalytic hydrolysis was carried out in 9 h cycles, mimicking sunrise/sunset, emulating natural solar radiation–for improved sustainability. As a drawback, this relatively short time of light exposure led to a low yield of lactide. Naturally, the yield can be enhanced through the use of more lamps with higher power outputs.

Powder X-ray diffraction confirmed that α-Fe_2_O_3_ was prepared as single-phase (Figure S1: Supporting Information). α-Fe_2_O_3_ has calculated structural parameters of *a* = 5.0332(3) Å, *b* = 5.0332(3) Å, *c* = 13.744(1) Å, α = β = 90°, γ = 120°, and the volume of the cell was 202.99 Å^3^. This was calculated based on a model by Pailhe et al.^22^ The band gap of α-Fe_2_O_3_ was calculated from solid UV–vis (eq 2 in the Supporting Information) as 2.20 eV, in close agreement with the value reported in the literature 2.60 eV.^21,22^ Scanning electron microscopy (SEM) was used to investigate the surface structure of the Fe_2_O_3_/PLA composite. The surface profile was found to be heavily distorted due to the reforming process of the PLA in the presence of the α-Fe_2_O_3_ nanoparticles, this is shown in Figure S2. It was measured that the average Fe crystalite size was 373.2 ± 137.7 nm; this means that they are not in the region of being considered as toxic. Additionally, the large deviation in particle size is accounted for by particle agglomeration that is present across the sample. Here, measured particles were as low as 138 nm and as high as 744 nm due to particle–particle aggregates. The SEM image (Figure S2) shows that the Fe_2_O_3_ is dispersed across the whole sample and not isolated to specific regions.

Gel permeation chromatography of the as received PLA, dissolved in DCM, showed that the number-average molar mass (*M*_n_) = 372 705 Da, the weight-average molar mass (*M*_w_) = 1.385 × 10^6^, and the *z*-average molar mass (*M*_*z*_).

**Figure 2:**
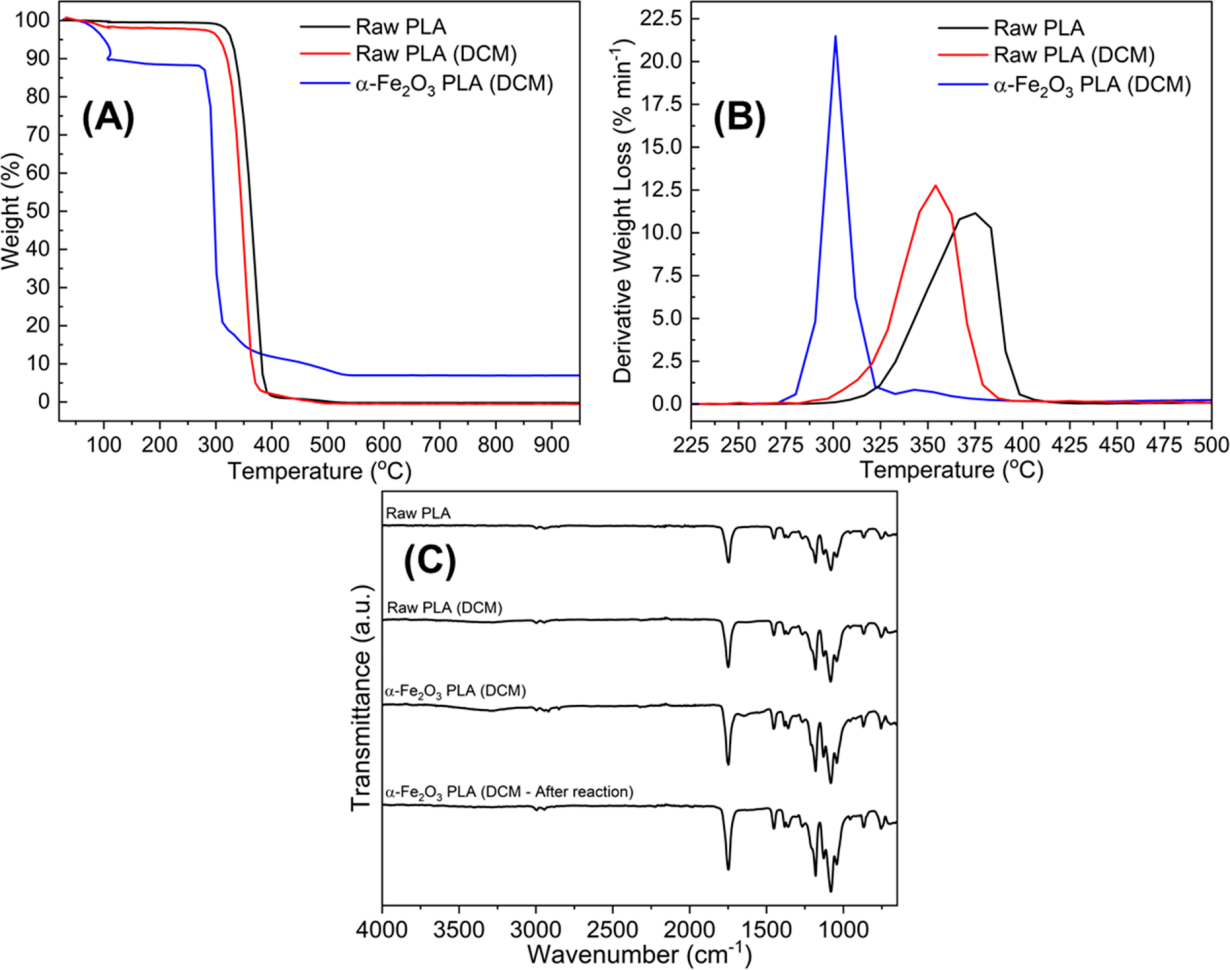
(a) Thermogravimetric analysis of raw PLA and Fe composite; (b) derivative weight loss profile; (c) FTIR spectra of PLA and α-Fe_2_O_3_/PLA composites before and after UV light exposure.

**Figure 3:**
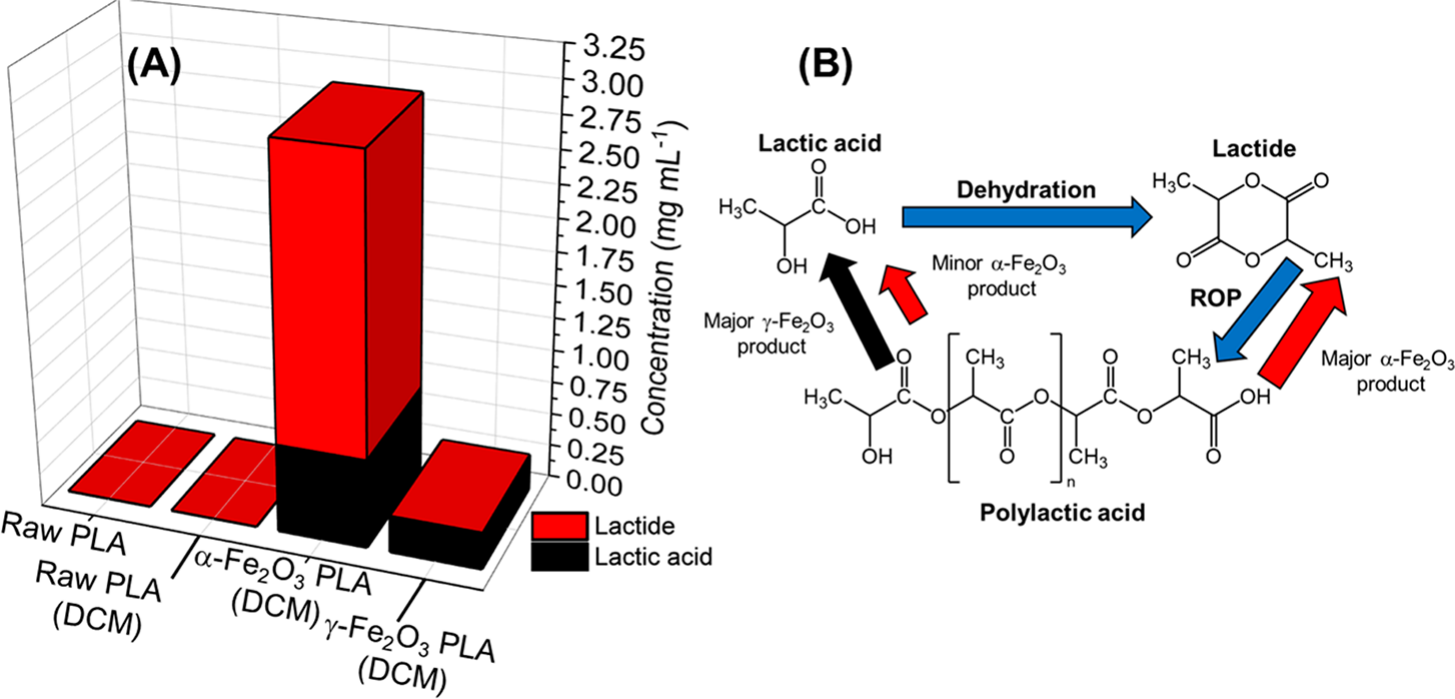
(a) Photocatalytic reaction selectivity after 90 h for both raw PLA and PLA composites. (b) Proposed reaction schematic with catalyst assignment (blue arrows denote reaction pathways; black arrow denotes γ-Fe_2_O_3_ selectivity, and red arrows denote α-Fe_2_O_3_ reaction selectivity).

**Figure 4:**
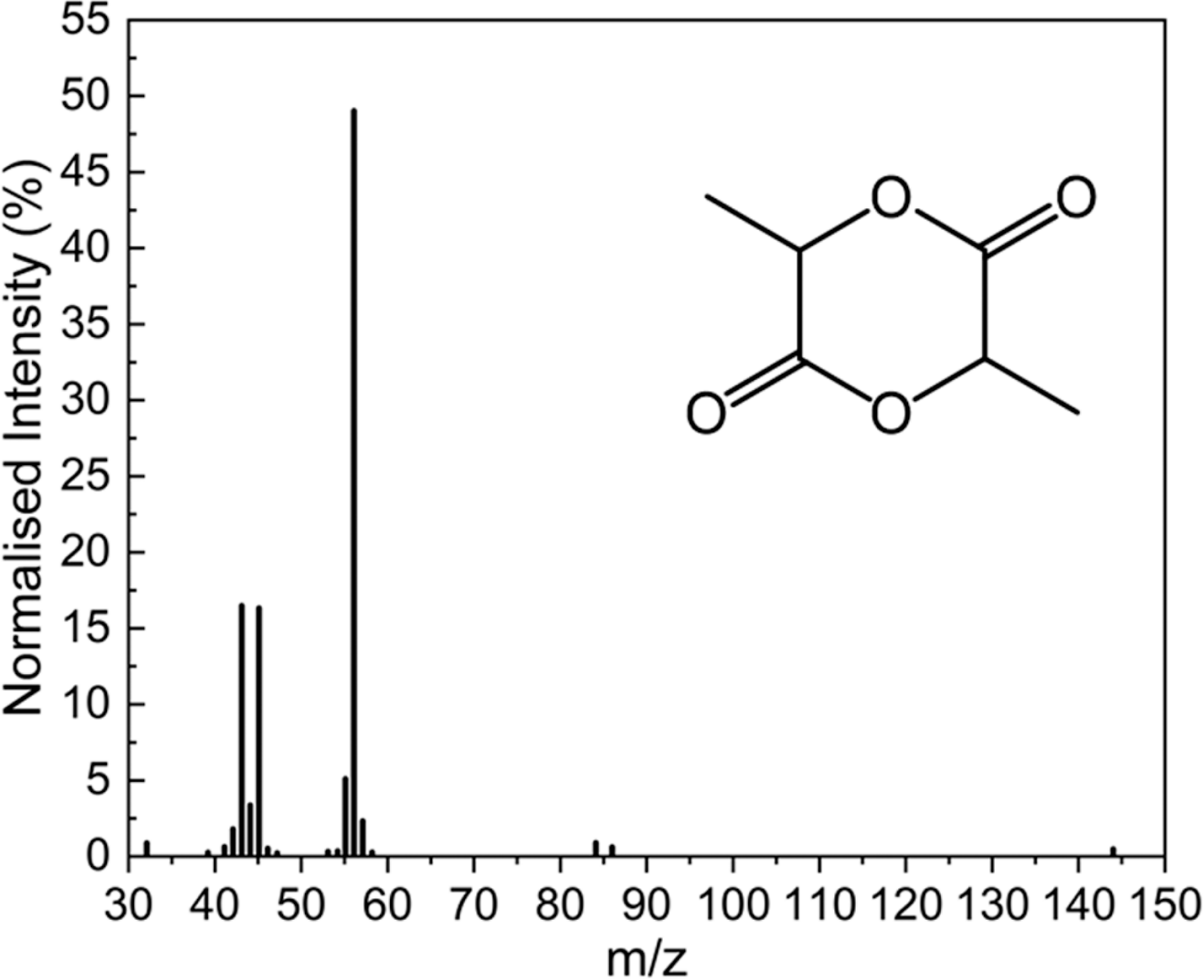
GCMS chromatogram of the product of photocatalytic hydrolysis of PLA, with the main peaks showing the presence of lactide.

The α-Fe_2_O_3_/PLA composite underwent thermogravimetric analysis along with raw PLA and reformed PLA after being dissolved in DCM; the raw PLA decomposed at 373.5 °C, reformed PLA at 353.4 °C and α-Fe_2_O_3_/PLA at 301.4 °C (Figure 2a and b). The differences in temperatures are due to the fact that physically the PLA structure has been altered, and the presence of embedded α-Fe_2_O_3_ particles has weakened the overall superstructure leading to decompositon at a lower temperature (Figure 2b). However, PLA is chemically unchanged, as shown by the comparison of the FTIR spectra (Figure 2c). Figure 2a indicates a mild weight loss for the raw PLA (DCM) and the α-Fe_2_O_3_/PLA composite; this is the presence of trapped DCM within the polymeric structure opposed to being adsorbed to the surface, as Figure 2c shows no C–Cl bonding (850–600 cm^–1^). Although there is evidence of catalytic conversion (Figures 3 and 4), there is no visible alteration to the chemical structure of the bulk α-Fe_2_O_3_/PLA composite after reaction (Figure 2c), which means that the composite is fragmenting over time as the photocatalytic hydrolysis takes place. Additionally, Table 1 shows the measured α-Fe_2_O_3_ content in the composite as a function of ash. Due to raw PLA containing only C, H, and O, the increase in ash for the composite is a surefire measure of photocatalyst loading (Fe_2_O_3_ = 6.91 wt%).

**Table 1:** Proximate and Ultimate Analysis of Raw PLA, PLA after Solubilization, and α-Fe_2_O_3_/PLA Composite

material	moisture (wt %)	volatile (wt %)	fixed carbon (wt %)	ash (wt %)	C (%)	H (%)	N (%)	O (%)^a^
raw PLA	0.19	99.81	0.00	0.00	49.7	5.7	0.0	44.6
raw PLA (DCM)	1.11	98.89	0.00	0.00	46.2	5.4	0.0	48.4
α-Fe_2_O_3_/PLA (DCM)	10.05	83.03	0.01	6.91	40.7	4.7	0.0	47.7

a Oxygen content of the raw material and composites is calculated by adding the C, H, N and Ash content together and subtracting from 100.

After irradiating the raw PLA, reformed PLA, and α-Fe_2_O_3_/PLA composite for 90 h, we deduced through HPLC that there was no photocatalytic reaction in samples without Fe (Figure 3a). However, by utilizing α-Fe_2_O_3_ embedded into the plastic matrix, we observe clear concentrations of lactide and lactic acid. The schematic in Figure 3b shows that a two stage process is taking place as lactic acid is being formed through the cleaving of the parent polymer, and a dehydration reaction is taking place which produces lactide in larger quanitities.

As a benchmark, we trialled the use of an alternative Fe_2_O_3_ polymorph (γ-Fe_2_O_3_), embedded in PLA using the same procedure. This material was also found to be active but only faciliiated the polymer cleaving step, not the following dehydration, making lactic acid the major product. However, due to lactic acid being the less desirable product and the low yield, this composite was not investigated further at this time. Lactide deriving from the decomposition of α-Fe_2_O_3_/PLA (DCM) was 2.21 mg/mL (9.5 wt%, from a composite of approximate mass 30 mg), indicating a reaction conversion of 32%. The presence of lactide was confirmed by GCMS after the sample was extracted with chloroform. Figure 4 shows the GCMS trace taken of the post α-Fe_2_O_3_/PLA composite reaction, after exposure to UV light for 90 h. This data was compared with the mass spectrum of lactide reported on the NIST Standard Reference Database.^25^ The comparison shows that the major product of the controlled photocatalytic hydrolysis is indeed 1,4-dioxane-2,5-dione, 3,6-dimethyl (lactide). The GCMS chromatogram was collected above 30 *m*/*z*, unlike the reference spectrum, which omitted the large CO signal at *m*/*z* 28. In addition to the lactide major product, GCMS also identified non-quantifiable secondary products: propanoic acid, butanoic acid and acetaldehyde.

As stated before, the photocatalytic decomposition of PLA proposed in this work derives from the pairing of two decomposition processes that PLA undergoes, hydrolysis and photolysis, in the presence of a photocatalyst. However, neither the hydrolysis nor the photolysis process directly led to lactide as the major product.

The hydrolysis reaction of PLA leads to the formation of lactic acid as well as other organic acids.^23^
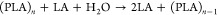
PLA has been previously reported to decompose when exposed to high intensity ultraviolet radiation photolysis.^24^ The process of photodegradation for aliphatic polyesters with carbonyl groups, like PLA, is initiated by photoionization on the carbonyl group (Norrish Type I, UV radiation around 220–280 nm), leading to a n−π* electron transition; this is followed by polymer chain scission (Norrish Type II).^25,26^ Several mechanisms have been reported; however, not one of them involves the formation of lactide.^27−29^ The formation of lactide has been observed previously during decomposition of PLA via thermolysis in vacuum.^24^ Thermolysis electrolysis is an energy intensive approach because it requires an alternating current of electricity to generate heat. Currently, due to only operating at a small scale, it is not practical for real world waste management. This is mimicked by previous work on the use of microwave technologies to upgrade PLA waste at 150 °C in an array of chemical components, increasing the cost of the PLA transformation process.^28,30,31^ Microwave technologies for waste management have been found to be energy inefficient, especially when operating at an isocratic, low temperature.^32,33^

For practical valorization of waste streams, the use of photocatalysis, albeit slow in the presented work, is a sustainable and selective approach to combat against plastic waste. This allows ambient temperature to be used, a lower net carbon process compared to thermally driven reactions such as previous work that utilized a homogeneous Zn acetate catalyst at ∼200 °C and ∼6 mbar in ethylene glycol.^34^

Modeling of possible mechanisms for the reaction from PLA to lactide is necessary for a complete understanding, especially when investigating the role of an embedded photocatalyst. However, it is reasonable to infer that the photocatalyst is the controlling element that directs the reaction toward the formation of the lactide, through a dehydration step. The photocatalytic process is based on the photogeneration of electrons and hole pairs within the photocatalyst. The electron–hole pair then generates hydroxyl radicals or directs electron transfer in the surrounding environment, in this case, the PLA in which the photocatalyst is embedded. Either the creation of radicals or the electron transfer is the additional element of photocatalytic hydrolysis compared to the processes of hydrolysis and photolysis. Hydrolysis in most cases leads to lactic acid and not lactide. The photodegradation of PLA is less clear, and several mechanisms have been reported, but again, none of them include the formation of lactide. Lactide is reported to be one of the products in some high energy thermal decomposition processes such as pyrolysis; however, due to the nature of this reaction, product selectivity can be variable.^28^ Hence, the formation of lactide has to be due to the presence of the additional radical formation or electron transfer from the photocatalyst, and can target the polymer due to the close proximity between the plastic itself and the photocatalyst within the α-Fe_2_O_3_/PLA matrix.

## Conclusions

The aim of this work was to prove the concept that combining hydrolysis and photolysis of PLA could be controlled, leading to a sustainable and environmentally friendly approach to upcycle plastic into value-added molecules, identified in the path to PLA decomposition.

Separately, photolysis and hydrolysis have been reported as sustainable decomposition routes for PLA. We brought the two processes together into a “one-pot reaction” under controlled conditions via the addition of a photocatalyst, coining the concept of photocatalytic hydrolysis. This was conduced by dissolving PLA ribbon in DCM and mixing with photoactive α-Fe_2_O_3_ nanoparticles, generating a composite material as the DCM is driven off. The composite material was then immersed in water and subjected to UV light.

Photocatalytic hydrolysis of PLA leads to the production of lactide and lactic acid, depending on the embedded Fe polymorph, where the alpha phase favors lactide (two step reaction) and the gamma phase favors lactic acid (single step, polymer cleaving) after 90 h utilizing a single low-powered UV source (25 W). Lactide is the main monomer used to prepare PLA via ring opening polymerization (ROP). However, its production is a long and an energy consuming process. Here, we show proof of concept that photocatalytic hydrolysis can be a greener approach to recycle PLA into lactide using only water, light, and iron oxide, utilizing less energy and as a result less carbon intensive than technologies such as pyrolysis. Our communicated method presents a faster route to PLA transformation than other lower carbon emitting processes, such as composting.

## Supporting Information Available

Experimental procedures including α-Fe_2_O_3_ particles and PLA composites; characterization used with instrument information; PXRD diffractogram of the α-Fe_2_O_3_; equations used; SEM image of the PLA composite; catalytic testing protocols with analysis information (PDF)
