# Supplementary material for: Photocatalytic Hydrolysis—A Sustainable Option for the Chemical Upcycling of Polylactic Acid
Source: ACS Environ Au. 2023 Oct 2;3(6):342–7. doi: 10.1021/acsenvironau.3c00040 (PMC10655588; doi:10.1021/acsenvironau.3c00040)
Supplement: Supplementary file 1 — vg3c00040_si_001.pdf [file vg3c00040_si_001.pdf]

Electronic Supporting Information

Photocatalytic Hydrolysis – A Sustainable Option for the Chemical Upcycling of Polylactic Acid

Antonia Garratt[a, b] Klaudia Nguyen[a, b], Alexander Brooke[a], Martin J. Taylor[c] and Maria Grazia Francesconi[a]*

[a] School of Natural Sciences, Chemistry, University of Hull, Cottingham Road, Hull, HU6 7RX, United Kingdom.

[b] Energy and Environment Institute, University of Hull, Cottingham Road, Hull, HU6 7RX, United Kingdom.

[c] School of Engineering, Chemical Engineering, University of Hull, Cottingham Road, Hull, HU6 7RX, United Kingdom.

EXPERIMENTAL

Preparation

Synthesis of α-Fe_2O_3 particles

Iron(II) Oxalate (0.4976 g, 3.11×10^{-3} mol) (Sigma-Aldrich, 99%) was heated at 500 °C at 1.0 °C per minute, holding for 4 h under static air environment using a Carbolite CWF 1200 furnace.

Preparation of α-Fe_2O_3/PLA composite

PLA Ribbon (RS PRO – PLA 1.75 mm) (0.7 g) was dissolved in 25 mL dichloromethane (VWR Chemicals, ≥99.8%) with the addition of sonication using a Ultrawave U100H PACKED sonicator for 1 hour to dissolve PLA fully. α-Fe_2O_3 powder (0.07g) was added gradually into the solution under continuous to stirring producing a 10:1 PLA/photocatalyst ratio by weight. The mixture was added dropwise onto a glass slide placed in a Ossila spin coater whilst spinning at intervals of 200 rpm for 1000 seconds. This process was repeated until a composite of approximately 30 mg was produced.

Characterisation

Gel Permeation Chromatography

Gel permeation Chromatography was carried out to determine the average molar mass of the PLA ribbon using a Viscotek with a VE 3580 RI detector, VE 1122 solvent delivery system, VE 5111 injector valve bracket and 270 dual detector. Here, 5 mg of PLA was dissolved in THF extra dry (Sigma-Aldrich, ≥99.9%) and injected.

Thermogravimetric Analyses (TGA)

Proximate analysis of the raw PLA and composites was carried out using a LECO 701 thermogravimetric analyser at~1.00 g scale where moisture, devolatilization and ash were measured. The proximate analysis method used was as follows: ambient to 107°C at a rate of 3°C/min under nitrogen, holding for 15 min before heating from 107°C to 950°C at 5°C/min, holding for 7 min before cooling to 600°C. This was followed by an ashing phase in air from 600 °C to 750°C at 3°C/min, before cooling to ambient conditions. Fixed carbon was calculated by subtracting the final ash mass from the sample mass before combustion.

Elemental Analyses

Elemental analysis (CHN) of all samples was acquired using a LECO Truspec Combustion analyser using sample sizes of 50.00-70.00 mg.

Fourier Transform Infrared (FTIR)

Fourier Transform Infrared (FTIR) spectra were obtained using a Thermo Scientific Nicolet iS5 with a PIKE MIRacle single reflection horizontal ATR accessory.

Powder X-Ray Diffraction (PXRD)

Powder X-ray Diffraction (PXRD) diffractograms were acquired using a Panalytical Empyrean diffractometer with Cu Kα radiation (λ = 0.154 nm). Analysis of the diffractograms was performed in HighScore Plus (2013, PANalytical B.V.) with the ICDD's PDF-2 2012 database to ensure that the photocatalysts obtained were single phase and contained no impurities.

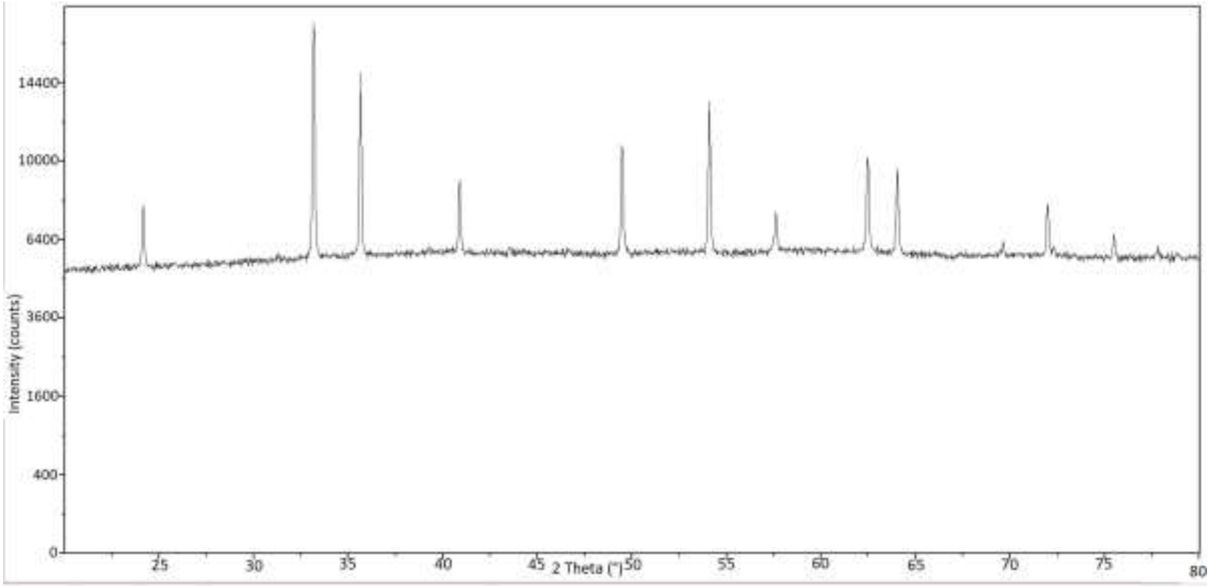

Figure S1: PXRD patterns for the α-Fe_2O_3 photocatalyst

UV-VIS Near Infrared spectroscopy (NIR)

UV-VIS Near Infrared spectroscopy (NIR) was used to determine the band gap of the photocatalysts, and barium sulphate ($BaSO_4$) was used as the reference material. Samples were diluted in $BaSO_4$ to a ratio of 1:2 of $BaSO_4$: Fe_2O_3. A Cary 5e Varian UV-VIS NIR spectrophotometer was used to obtain a diffuse UV-VIS spectrum and data was recorded in the range of 200 – 900 nm. The band gap can be calculated from the diffuse spectra by normalising the spectra against the background using the Kubelka – Munk function (**Equation 2**). The Tauc plot was drawn, where the Kubelka -Munk function was plotted against electron volts, which

were calculated from given wavelength using **Equation 1**. A tangent is then drawn to determine the accurate interception of the x-axis to obtain the band gap for the photocatalyst.[10, 11]

$E(J) = \frac{hc}{\lambda}$ (Equation 1)

$F\ (R)\infty = \frac{K}{S} = \frac{(1-R)^2}{2R\infty}$ (Equation 2)

where h is Planck's constant, c is the speed of light and λ is the wavelength. The energy can be converted to electron volts by multiplying by $6.242 \times 10^{18}\ eV$ to obtain the energy band gap between the valence and conduction bands.

Scanning Electron Microscopy (SEM)

Scanning electron microscopy was completed using a Zeiss EVO 60 at 10-2 Pa with a 20 kV electron acceleration voltage. Samples were adhered to a conductive carbon tape and Au coated to increase image contrast.

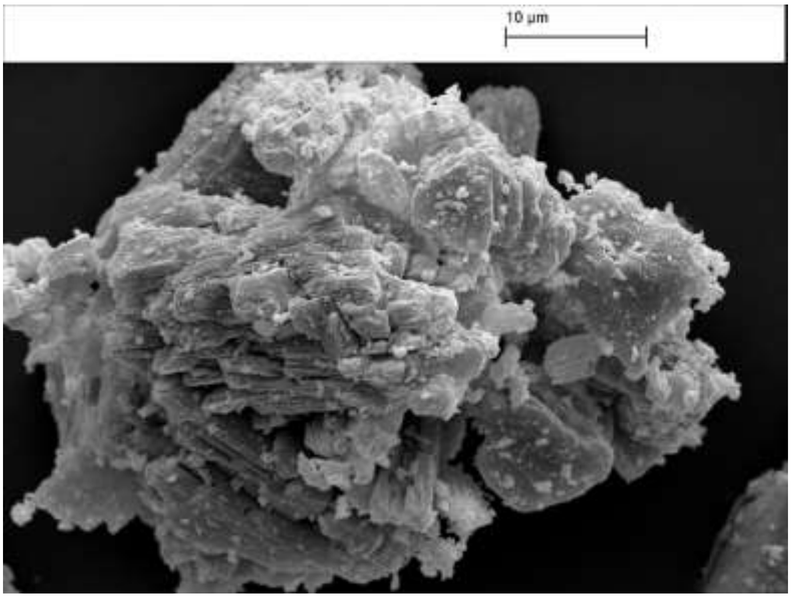

Figure S2: SEM image of the α-Fe_2O_3/PLA composite at a magnification of 5000x

Catalytic Testing and Analysis

The α-Fe_2O_3/PLA composite (~0.03 g) was put in a quartz cuvette (10mm path length), which was the filled with 3.5 mL distilled water. The cuvette was exposed to UV light (258 nm, 25 W) for 90 hours. Subsequent analysis of aliquots were extracted with chloroform (Thermo Fischer Scientific, ≥99.5%) using an Agilent 5973 Gas Chromatograph with Mass Spectrometer detector (GCMS) outfitted with a HP 5ms column. Quantitative analysis for lactic acid and lactide was acquired using a Perkin Elmer 200 HPLC with a 785 UV/Vis detector, calibration plots for lactide and lactic acid are shown in Figure S3. The analysis was performed with a mobile phase solution of 1% phosphoric acid (Alfa Aesar, 85%), 10% acetonitrile in water (Riedel-de-Haen, HPLC grade) through a Phenomex Luna 5u C18 100A column (150 x 4.60 mm 5 micron). The UV detector was set to 210 nm for lactic acid and 250 nm for lactide.

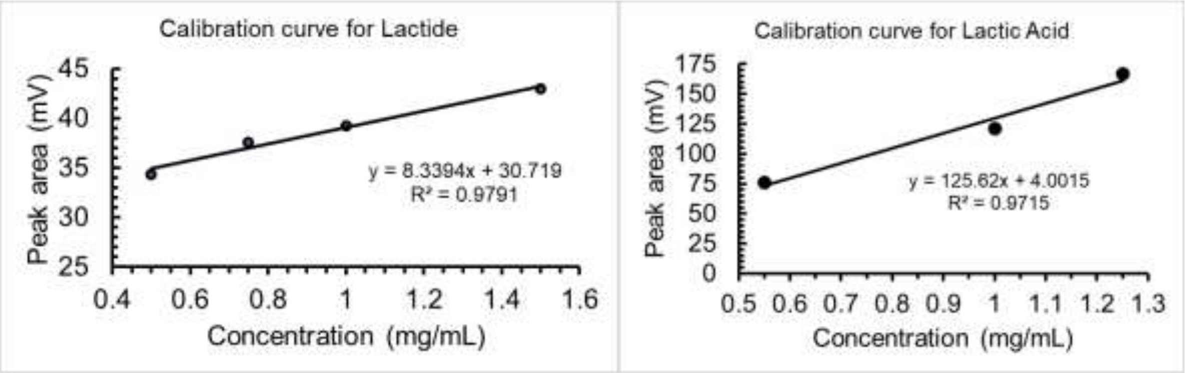

Figure S3: HPLC calibration data for lactide and lactic acid
